# Dibromido{2-[2-(piperidinium-1-yl)ethyl­imino­meth­yl]phenolato}zinc(II) monohydrate

**DOI:** 10.1107/S1600536808015730

**Published:** 2008-06-07

**Authors:** Yi-Jun Wei, Feng-Wu Wang, Qi-Yong Zhu

**Affiliations:** a Department of Chemistry, Huainan Normal College, Huainan 232001, People’s Republic of China

## Abstract

The asymmetric unit of the title compound, [ZnBr_2_(C_14_H_20_N_2_O)]·H_2_O, consists of a mononuclear Schiff base zinc(II) complex mol­ecule and a solvent water mol­ecule. The Zn^II^ atom is four-coordinated in an approximately tetra­hedral geometry, binding to the imine N and phenolate O atoms of the neutral zwitterionic Schiff base ligand and to two terminal Br^−^ anions. In the crystal structure, mol­ecules are linked through inter­molecular O—H⋯Br and O—H⋯O hydrogen bonds, forming chains running along the *b* axis.

## Related literature

For the background to Schiff base zinc(II) complexes, see: Bhosekar *et al.* (2006 ▶); Chisholm *et al.* (2001 ▶); Jian *et al.* (2004 ▶); Lacroix *et al.* (1996 ▶); Tatar *et al.* (2002 ▶). For related structures, see: Ma, Gu *et al.* (2006 ▶); Ma, Lv *et al.* (2006 ▶); Peng & Hou (2006 ▶); Peng *et al.* (2006 ▶); Wei *et al.* (2007 ▶); Zhang *et al.* (2008 ▶); Zhu *et al.* (2007 ▶).
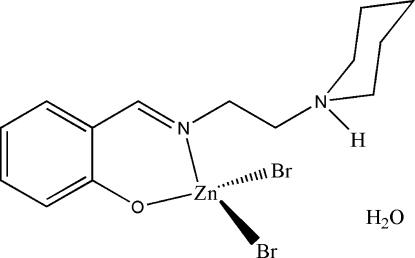


## Experimental

### 

#### Crystal data

[ZnBr_2_(C_14_H_20_N_2_O)]·H_2_O
*M* *_r_* = 475.53
Triclinic, 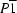

*a* = 9.2997 (18) Å
*b* = 10.1776 (17) Å
*c* = 11.1667 (18) Å
α = 71.510 (2)°
β = 71.215 (2)°
γ = 67.571 (2)°
*V* = 901.8 (3) Å^3^
*Z* = 2
Mo *K*α radiation
μ = 5.80 mm^−1^
*T* = 298 (2) K
0.20 × 0.20 × 0.18 mm

#### Data collection

Bruker SMART 1000 CCD area-detector diffractometer
Absorption correction: multi-scan (*SADABS*; Sheldrick, 1996 ▶) *T*_min_ = 0.390, *T*_max_ = 0.422 (expected range = 0.326–0.352)
5468 measured reflections
3983 independent reflections
2891 reflections with *I* > 2σ(*I*)
*R*_int_ = 0.016

#### Refinement

*R*[*F* ^2^ > 2σ(*F* ^2^)] = 0.037
*wR*(*F* ^2^) = 0.091
*S* = 1.02
3983 reflections
199 parameters
4 restraints
H atoms treated by a mixture of independent and constrained refinement
Δρ_max_ = 0.72 e Å^−3^
Δρ_min_ = −0.55 e Å^−3^

### 

Data collection: *SMART* (Bruker, 2002 ▶); cell refinement: *SAINT* (Bruker, 2002 ▶); data reduction: *SAINT*; program(s) used to solve structure: *SHELXS97* (Sheldrick, 2008 ▶); program(s) used to refine structure: *SHELXL97* (Sheldrick, 2008 ▶); molecular graphics: *SHELXTL* (Sheldrick, 2008 ▶); software used to prepare material for publication: *SHELXTL*.

## Supplementary Material

Crystal structure: contains datablocks global, I. DOI: 10.1107/S1600536808015730/sj2508sup1.cif

Structure factors: contains datablocks I. DOI: 10.1107/S1600536808015730/sj2508Isup2.hkl

Additional supplementary materials:  crystallographic information; 3D view; checkCIF report

## Figures and Tables

**Table:** 

Zn1—O1	1.936 (2)
Zn1—N1	2.024 (3)
Zn1—Br1	2.3417 (7)
Zn1—Br2	2.3991 (7)

**Table:** 

O1—Zn1—N1	93.91 (11)
O1—Zn1—Br1	116.12 (8)
N1—Zn1—Br1	113.04 (8)
O1—Zn1—Br2	109.78 (8)
N1—Zn1—Br2	108.84 (9)
Br1—Zn1—Br2	113.42 (2)

**Table 2:** Hydrogen-bond geometry (Å, °)

*D*—H⋯*A*	*D*—H	H⋯*A*	*D*⋯*A*	*D*—H⋯*A*
N2—H2⋯O2	0.90 (4)	1.89 (4)	2.777 (4)	169 (4)
O2—H2*A*⋯Br2^i^	0.85 (4)	2.57 (4)	3.399 (3)	165 (4)
O2—H2*B*⋯O1^ii^	0.86 (3)	1.93 (4)	2.762 (4)	165 (5)

Symmetry codes: (i) 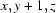; (ii) 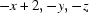.

**Fig. 1.**
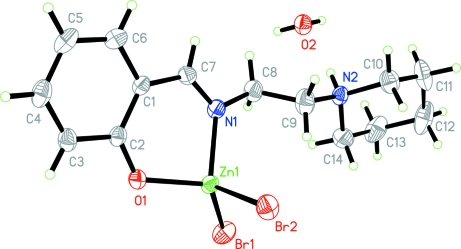
The molecular structure of (I), shown with 30% probability displacement ellipsoids.

**Fig. 2.**
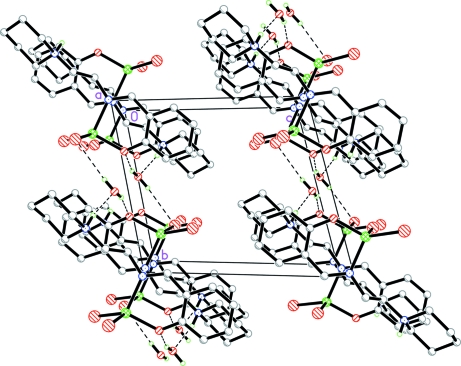
Molecular packing of (I), viewed along the a axis. Intermolecular hydrogen bonds are shown as dashed lines.

## supplementary crystallographic information

### Comment

Zinc(II) complexes derived from Schiff base ligands have been studied extensively due to their interesting structures and wide applications (Lacroix *et al.*, 1996; Chisholm *et al.*, 2001; Jian *et al.*, 2004; Tatar *et al.*, 2002; Bhosekar *et al.*, 2006). Recently, we have reported two Schiff base zinc(II) complexes with bromide ligands (Wei *et al.*, 2007; Zhu *et al.*, 2007). As a continuation of our work on the structures of such complexes, we report herein the crystal structure of the new title complex, (I), which is isostructural with the zinc(II) complex with chloride ligands (Zhang *et al.*, 2008).

The tetrahedral coordination sphere of Zn^II^ atom in (I) is formed by the imine N and phenolate O atoms of the Schiff base ligand and by two terminal Br^-^ anions (Fig. 1). The coordinate bond distances (Table 1) are typical and comparable with the values in other similar zinc(II) complexes (Peng & Hou, 2006; Peng *et al.*, 2006; Ma, Gu *et al.*, 2006; Ma, Lv *et al.*, 2006). The O1—Zn1—N1 and O1—Zn1—Br1 bond angles deviate most from ideal tetrahedral geometry with values of 93.91 (11) and 116.12 (8)°, respectively. The other angles in the coordination sphere are in the range 108.84 (9)–113.42 (2)° (Table 1).

In the crystal structure of (I), molecules are linked through intermolecular O—H···Br and O—H···O hydrogen bonds (Table 2), forming chains running along the *b* axis (Fig. 2).

### Experimental

Compound (I) was obtained by stirring of salicylaldehyde (0.1 mmol, 12.2 mg), 2-piperidin-1-ylethylamine (0.1 mmol, 12.8 mg), and zinc(II) bromide (0.1 mmol, 22.5 mg) in methanol (20 ml) for 30 min at room temperature. The reaction mixture was fitered. Yellow block-shaped single crystals suitable for X-ray diffraction formed from the filtrate after one day.

### Refinement

Atoms H2, H2A and H2B were located in a difference Fourier map and refined isotropically, with the N—H, O—H, and H···H distances restrained to 0.90 (1), 0.85 (1), and 1.37 (2) Å, respectively. Other H atom positions were positioned geometrically (C—H = 0.93–0.97 Å) and refined as riding, with *U*_iso_(H) values set at 1.2*U*_eq_(C).

### Crystal data

**Table:**

[ZnBr_2_(C_14_H_20_N_2_O)]·H_2_O	*Z* = 2
*M**_r_* = 475.53	*F*_000_ = 472
Triclinic, *P*1	*D*_x_ = 1.751 Mg m^−^^3^
Hall symbol: -P 1	Mo *K*α radiation λ = 0.71073 Å
*a* = 9.2997 (18) Å	Cell parameters from 1797 reflections
*b* = 10.1776 (17) Å	θ = 2.2–25.4º
*c* = 11.1667 (18) Å	µ = 5.80 mm^−^^1^
α = 71.510 (2)º	*T* = 298 (2) K
β = 71.215 (2)º	Block, yellow
γ = 67.571 (2)º	0.20 × 0.20 × 0.18 mm
*V* = 901.8 (3) Å^3^	

### Data collection

**Table:**

Bruker SMART 1000 CCD area-detector diffractometer	3983 independent reflections
Radiation source: fine-focus sealed tube	2891 reflections with *I* > 2σ(*I*)
Monochromator: graphite	*R*_int_ = 0.016
*T* = 298(2) K	θ_max_ = 27.5º
ω scans	θ_min_ = 2.0º
Absorption correction: multi-scan(SADABS; Sheldrick, 1996)	*h* = −12→9
*T*_min_ = 0.390, *T*_max_ = 0.422	*k* = −13→12
5468 measured reflections	*l* = −14→14

### Refinement

**Table:**

Refinement on *F*^2^	Secondary atom site location: difference Fourier map
Least-squares matrix: full	Hydrogen site location: inferred from neighbouring sites
*R*[*F*^2^ > 2σ(*F*^2^)] = 0.037	H atoms treated by a mixture of independent and constrained refinement
*wR*(*F*^2^) = 0.091	*w* = 1/[σ^2^(*F*_o_^2^) + (0.0405*P*)^2^ + 0.3508*P*] where *P* = (*F*_o_^2^ + 2*F*_c_^2^)/3
*S* = 1.03	(Δ/σ)_max_ = 0.001
3983 reflections	Δρ_max_ = 0.72 e Å^−^^3^
199 parameters	Δρ_min_ = −0.55 e Å^−^^3^
4 restraints	Extinction correction: none
Primary atom site location: structure-invariant direct methods	

### Special details

**Table:**

Geometry. All e.s.d.'s (except the e.s.d. in the dihedral angle between two l.s. planes) are estimated using the full covariance matrix. The cell e.s.d.'s are taken into account individually in the estimation of e.s.d.'s in distances, angles and torsion angles; correlations between e.s.d.'s in cell parameters are only used when they are defined by crystal symmetry. An approximate (isotropic) treatment of cell e.s.d.'s is used for estimating e.s.d.'s involving l.s. planes.
Refinement. Refinement of *F*^2^ against ALL reflections. The weighted *R*-factor *wR* and goodness of fit *S* are based on *F*^2^, conventional *R*-factors *R* are based on *F*, with *F* set to zero for negative *F*^2^. The threshold expression of *F*^2^ > σ(*F*^2^) is used only for calculating *R*-factors(gt) *etc*. and is not relevant to the choice of reflections for refinement. *R*-factors based on *F*^2^ are statistically about twice as large as those based on *F*, and *R*- factors based on ALL data will be even larger.

### Fractional atomic coordinates and isotropic or equivalent isotropic displacement parameters (Å^2^)

**Table:**

	*x*	*y*	*z*	*U*_iso_*/*U*_eq_	
Zn1	0.82333 (5)	−0.20793 (4)	0.11443 (4)	0.03909 (13)	
Br1	0.93736 (6)	−0.24561 (5)	0.28619 (4)	0.06059 (15)	
Br2	0.56849 (5)	−0.24885 (5)	0.18395 (4)	0.05682 (14)	
O1	0.9582 (3)	−0.3089 (3)	−0.0226 (2)	0.0451 (6)	
O2	0.7190 (3)	0.4568 (3)	0.0481 (3)	0.0515 (7)	
N1	0.8013 (4)	−0.0074 (3)	−0.0039 (3)	0.0406 (7)	
N2	0.5644 (4)	0.2574 (3)	0.2120 (3)	0.0420 (7)	
C1	0.8738 (4)	−0.1069 (4)	−0.1951 (3)	0.0378 (8)	
C2	0.9415 (4)	−0.2555 (4)	−0.1434 (3)	0.0379 (8)	
C3	0.9993 (5)	−0.3518 (4)	−0.2277 (4)	0.0451 (9)	
H3	1.0451	−0.4508	−0.1960	0.054*	
C4	0.9897 (5)	−0.3031 (5)	−0.3550 (4)	0.0555 (11)	
H4	1.0283	−0.3696	−0.4077	0.067*	
C5	0.9234 (6)	−0.1561 (5)	−0.4069 (4)	0.0594 (12)	
H5	0.9176	−0.1236	−0.4935	0.071*	
C6	0.8668 (5)	−0.0600 (5)	−0.3273 (4)	0.0513 (10)	
H6	0.8225	0.0387	−0.3611	0.062*	
C7	0.8178 (4)	0.0091 (4)	−0.1258 (4)	0.0409 (8)	
H7	0.7915	0.1043	−0.1745	0.049*	
C8	0.7581 (5)	0.1241 (4)	0.0443 (4)	0.0552 (11)	
H8A	0.7560	0.2080	−0.0279	0.066*	
H8B	0.8374	0.1140	0.0884	0.066*	
C9	0.5982 (5)	0.1467 (5)	0.1357 (5)	0.0633 (12)	
H9A	0.5928	0.0547	0.1952	0.076*	
H9B	0.5166	0.1790	0.0870	0.076*	
C10	0.3889 (5)	0.3298 (5)	0.2454 (5)	0.0594 (11)	
H10A	0.3359	0.2573	0.2962	0.071*	
H10B	0.3502	0.3761	0.1664	0.071*	
C11	0.3486 (6)	0.4435 (6)	0.3223 (6)	0.0836 (17)	
H11A	0.3915	0.5216	0.2677	0.100*	
H11B	0.2334	0.4848	0.3476	0.100*	
C12	0.4153 (7)	0.3795 (7)	0.4409 (6)	0.094 (2)	
H12A	0.3925	0.4556	0.4853	0.113*	
H12B	0.3651	0.3077	0.4997	0.113*	
C13	0.5908 (6)	0.3095 (7)	0.4042 (5)	0.0835 (17)	
H13A	0.6325	0.2653	0.4819	0.100*	
H13B	0.6414	0.3830	0.3510	0.100*	
C14	0.6302 (6)	0.1951 (5)	0.3306 (5)	0.0701 (13)	
H14A	0.7453	0.1528	0.3059	0.084*	
H14B	0.5862	0.1179	0.3862	0.084*	
H2B	0.8210 (13)	0.423 (5)	0.029 (5)	0.080*	
H2A	0.699 (4)	0.524 (4)	0.086 (4)	0.080*	
H2	0.611 (5)	0.327 (4)	0.168 (4)	0.080*	

### Atomic displacement parameters (Å^2^)

**Table:**

	*U*^11^	*U*^22^	*U*^33^	*U*^12^	*U*^13^	*U*^23^
Zn1	0.0453 (3)	0.0381 (2)	0.0358 (2)	−0.01543 (19)	−0.00516 (18)	−0.01193 (18)
Br1	0.0684 (3)	0.0803 (3)	0.0459 (2)	−0.0337 (3)	−0.0178 (2)	−0.0131 (2)
Br2	0.0462 (3)	0.0559 (3)	0.0720 (3)	−0.0217 (2)	−0.0069 (2)	−0.0184 (2)
O1	0.0509 (16)	0.0409 (14)	0.0377 (14)	−0.0066 (12)	−0.0070 (12)	−0.0138 (11)
O2	0.0503 (17)	0.0479 (16)	0.0572 (18)	−0.0181 (14)	−0.0049 (14)	−0.0170 (13)
N1	0.0497 (19)	0.0346 (16)	0.0412 (17)	−0.0198 (14)	−0.0023 (14)	−0.0134 (13)
N2	0.0438 (19)	0.0411 (18)	0.0415 (17)	−0.0163 (15)	−0.0001 (14)	−0.0157 (14)
C1	0.041 (2)	0.041 (2)	0.0329 (18)	−0.0188 (17)	−0.0024 (15)	−0.0099 (15)
C2	0.033 (2)	0.044 (2)	0.0387 (19)	−0.0167 (16)	−0.0012 (15)	−0.0128 (16)
C3	0.046 (2)	0.044 (2)	0.049 (2)	−0.0169 (18)	−0.0028 (18)	−0.0200 (18)
C4	0.059 (3)	0.071 (3)	0.049 (2)	−0.030 (2)	0.001 (2)	−0.031 (2)
C5	0.071 (3)	0.081 (3)	0.034 (2)	−0.040 (3)	−0.002 (2)	−0.013 (2)
C6	0.057 (3)	0.054 (2)	0.042 (2)	−0.023 (2)	−0.0084 (19)	−0.0033 (18)
C7	0.039 (2)	0.0348 (19)	0.047 (2)	−0.0154 (16)	−0.0043 (17)	−0.0070 (16)
C8	0.064 (3)	0.043 (2)	0.059 (3)	−0.022 (2)	0.001 (2)	−0.0222 (19)
C9	0.061 (3)	0.063 (3)	0.076 (3)	−0.026 (2)	0.001 (2)	−0.037 (2)
C10	0.046 (3)	0.062 (3)	0.074 (3)	−0.018 (2)	−0.011 (2)	−0.022 (2)
C11	0.040 (3)	0.071 (3)	0.140 (5)	−0.011 (2)	0.002 (3)	−0.056 (4)
C12	0.083 (4)	0.141 (5)	0.084 (4)	−0.056 (4)	0.023 (3)	−0.073 (4)
C13	0.077 (4)	0.140 (5)	0.050 (3)	−0.045 (4)	−0.013 (3)	−0.030 (3)
C14	0.055 (3)	0.074 (3)	0.065 (3)	−0.011 (3)	−0.019 (2)	−0.002 (3)

### Geometric parameters (Å, °)

**Table:**

Zn1—O1	1.936 (2)	C5—H5	0.9300
Zn1—N1	2.024 (3)	C6—H6	0.9300
Zn1—Br1	2.3417 (7)	C7—H7	0.9300
Zn1—Br2	2.3991 (7)	C8—C9	1.488 (6)
O1—C2	1.321 (4)	C8—H8A	0.9700
O2—H2B	0.86 (3)	C8—H8B	0.9700
O2—H2A	0.85 (4)	C9—H9A	0.9700
N1—C7	1.282 (5)	C9—H9B	0.9700
N1—C8	1.467 (4)	C10—C11	1.519 (6)
N2—C10	1.487 (5)	C10—H10A	0.9700
N2—C14	1.491 (5)	C10—H10B	0.9700
N2—C9	1.503 (5)	C11—C12	1.496 (8)
N2—H2	0.90 (4)	C11—H11A	0.9700
C1—C2	1.404 (5)	C11—H11B	0.9700
C1—C6	1.415 (5)	C12—C13	1.485 (7)
C1—C7	1.454 (5)	C12—H12A	0.9700
C2—C3	1.411 (5)	C12—H12B	0.9700
C3—C4	1.370 (6)	C13—C14	1.502 (7)
C3—H3	0.9300	C13—H13A	0.9700
C4—C5	1.389 (6)	C13—H13B	0.9700
C4—H4	0.9300	C14—H14A	0.9700
C5—C6	1.371 (6)	C14—H14B	0.9700
			
O1—Zn1—N1	93.91 (11)	C9—C8—H8A	109.7
O1—Zn1—Br1	116.12 (8)	N1—C8—H8B	109.7
N1—Zn1—Br1	113.04 (8)	C9—C8—H8B	109.7
O1—Zn1—Br2	109.78 (8)	H8A—C8—H8B	108.2
N1—Zn1—Br2	108.84 (9)	C8—C9—N2	112.2 (3)
Br1—Zn1—Br2	113.42 (2)	C8—C9—H9A	109.2
C2—O1—Zn1	121.6 (2)	N2—C9—H9A	109.2
H2B—O2—H2A	105 (2)	C8—C9—H9B	109.2
C7—N1—C8	117.3 (3)	N2—C9—H9B	109.2
C7—N1—Zn1	119.8 (2)	H9A—C9—H9B	107.9
C8—N1—Zn1	122.8 (2)	N2—C10—C11	110.8 (3)
C10—N2—C14	111.0 (3)	N2—C10—H10A	109.5
C10—N2—C9	108.7 (3)	C11—C10—H10A	109.5
C14—N2—C9	113.6 (3)	N2—C10—H10B	109.5
C10—N2—H2	108 (3)	C11—C10—H10B	109.5
C14—N2—H2	102 (3)	H10A—C10—H10B	108.1
C9—N2—H2	113 (3)	C12—C11—C10	111.6 (4)
C2—C1—C6	119.5 (3)	C12—C11—H11A	109.3
C2—C1—C7	125.2 (3)	C10—C11—H11A	109.3
C6—C1—C7	115.1 (3)	C12—C11—H11B	109.3
O1—C2—C1	123.9 (3)	C10—C11—H11B	109.3
O1—C2—C3	118.6 (3)	H11A—C11—H11B	108.0
C1—C2—C3	117.4 (3)	C13—C12—C11	110.0 (4)
C4—C3—C2	121.6 (4)	C13—C12—H12A	109.7
C4—C3—H3	119.2	C11—C12—H12A	109.7
C2—C3—H3	119.2	C13—C12—H12B	109.7
C3—C4—C5	121.2 (4)	C11—C12—H12B	109.7
C3—C4—H4	119.4	H12A—C12—H12B	108.2
C5—C4—H4	119.4	C12—C13—C14	110.9 (4)
C6—C5—C4	118.4 (4)	C12—C13—H13A	109.5
C6—C5—H5	120.8	C14—C13—H13A	109.5
C4—C5—H5	120.8	C12—C13—H13B	109.5
C5—C6—C1	121.8 (4)	C14—C13—H13B	109.5
C5—C6—H6	119.1	H13A—C13—H13B	108.0
C1—C6—H6	119.1	N2—C14—C13	111.4 (4)
N1—C7—C1	126.2 (3)	N2—C14—H14A	109.4
N1—C7—H7	116.9	C13—C14—H14A	109.4
C1—C7—H7	116.9	N2—C14—H14B	109.4
N1—C8—C9	109.9 (3)	C13—C14—H14B	109.4
N1—C8—H8A	109.7	H14A—C14—H14B	108.0

### Hydrogen-bond geometry (Å, °)

**Table:**

*D*—H···*A*	*D*—H	H···*A*	*D*···*A*	*D*—H···*A*
N2—H2···O2	0.90 (4)	1.89 (4)	2.777 (4)	169 (4)
O2—H2A···Br2^i^	0.85 (4)	2.57 (4)	3.399 (3)	165 (4)
O2—H2B···O1^ii^	0.86 (3)	1.93 (4)	2.762 (4)	165 (5)

Symmetry codes: (i) *x*, *y*+1, *z*; (ii) −*x*+2, −*y*, −*z*.

## References

[bb1] Bhosekar, G., Jess, I. & Näther, C. (2006). *Acta Cryst.* E**62**, m2073–m2074.

[bb2] Bruker (2002). *SMART*, *SAINT* and *SHELXTL* Bruker AXS Inc., Madison, Wisconsin, USA.

[bb3] Chisholm, M. H., Gallucci, J. C. & Zhen, H. (2001). *Inorg. Chem.* **40**, 5051–5054. 10.1021/ic010560e 11531458

[bb4] Jian, F., Li, C., Sun, P. & Xiao, H. (2004). *Acta Cryst.* E**60**, m1811–m1812.

[bb5] Lacroix, P. G., Di Bella, S. & Ledoux, I. (1996). *Chem. Mater.* **8**, 541–545.

[bb6] Ma, J.-Y., Gu, S.-H., Guo, J.-W., Lv, B.-L. & Yin, W.-P. (2006). *Acta Cryst.* E**62**, m1437–m1438.

[bb7] Ma, J.-Y., Lv, B.-L., Gu, S.-H., Guo, J.-W. & Yin, W.-P. (2006). *Acta Cryst.* E**62**, m1322–m1323.

[bb8] Peng, S.-J. & Hou, H.-Y. (2006). *Acta Cryst.* E**62**, m2947–m2949.

[bb9] Peng, S.-J., Zhou, C.-S. & Yang, T. (2006). *Acta Cryst.* E**62**, m1413–m1415.

[bb10] Sheldrick, G. M. (1996). *SADABS* University of Göttingen, Germany.

[bb11] Sheldrick, G. M. (2008). *Acta Cryst.* A**64**, 112–122. 10.1107/S0108767307043930 18156677

[bb12] Tatar, L., Atakol, O. & Ülkü, D. (2002). *Acta Cryst.* E**58**, m83–m85.

[bb13] Wei, Y.-J., Wang, F.-W. & Zhu, Q.-Y. (2007). *Acta Cryst.* E**63**, m654–m655.

[bb14] Zhang, D.-F., Zhou, M.-H. & Yuan, C.-J. (2008). *Acta Cryst.* E**64**, m825–m826. 10.1107/S1600536808014311 PMC2961511 21202508

[bb15] Zhu, Q.-Y., Wei, Y.-J. & Wang, F.-W. (2007). *Acta Cryst.* E**63**, m1431–m1432.

